# Clinical-Scale Mesenchymal Stem Cell-Derived Extracellular Vesicle Therapy for Wound Healing

**DOI:** 10.3390/ijms24054273

**Published:** 2023-02-21

**Authors:** Jieun Kim, Eun Hee Kim, Hanbee Lee, Ji Hee Sung, Oh Young Bang

**Affiliations:** 1 Cell and Gene Institute, Samsung Medical Center, Seoul 06351, Republic of Korea; 2 Translational and Stem Cell Research Laboratory on Stroke, Samsung Medical Center, Seoul 06351, Republic of Korea; 3 S&E Bio, Inc., Seoul 05855, Republic of Korea; 4 Department of Neurology, Samsung Medical Center, Sungkyunkwan University School of Medicine, Seoul 06351, Republic of Korea

**Keywords:** mesenchymal stem cells, extracellular vesicles, exosomes, wound healing, functional recovery

## Abstract

We developed an extracellular vesicle (EV) bioprocessing platform for the scalable production of human Wharton’s jelly mesenchymal stem cell (MSC)-derived EVs. The effects of clinical-scale MSC-EV products on wound healing were tested in two different wound models: subcutaneous injection of EVs in a conventional full-thickness rat model and topical application of EVs using a sterile re-absorbable gelatin sponge in the chamber mouse model that was developed to prevent the contraction of wound areas. In vivo efficacy tests showed that treatment with MSC-EVs improved the recovery following wound injury, regardless of the type of wound model or mode of treatment. In vitro mechanistic studies using multiple cell lines involved in wound healing showed that EV therapy contributed to all stages of wound healing, such as anti-inflammation and proliferation/migration of keratinocytes, fibroblasts, and endothelial cells, to enhance wound re-epithelialization, extracellular matrix remodeling, and angiogenesis.

## 1. Introduction

Cutaneous wounds are common injuries caused by trauma, burns, ulcers, or surgery. Non-healing cutaneous wounds can impose severe clinical burdens on patients without effective treatment strategies. The beneficial effects of exogenous mesenchymal stem cells (MSCs) on wound healing have been observed in various animal models and clinical cases [1,2] Clinical test results using MSCs to enhance wound healing have been promising [3,4]. Notwithstanding the promising results obtained in clinical trials, MSC-based therapies are not considered a standard of care in clinical settings due to various limitations to their applicability [5,6].

A cell-free treatment paradigm using MSC-derived extracellular vesicles (EVs) can avoid the cell-related problems associated with stem cell therapy and exert the paracrine actions of MSCs. In addition, the “off-the-shelf” use of allogeneic MSC-derived EVs from healthy and young stem cells, such as MSCs derived from the umbilical cord, has the advantage of scalable production and storage with standardized procedures with high restorative capacity. However, critical hurdles remain in the translation of MSC-EVs into clinical therapeutics. Previous studies have used EV preparations obtained from the conventional 2D culture of MSCs; however, to date, no preclinical or clinical studies have examined the effects of MSC-EVs via scale-up production with customized therapeutic properties. We have previously reported that MSCs 3D-cultured as size-controlled cellular aggregates on a large scale better preserved the innate phenotype and properties of MSCs compared to 2D monolayer cultures, which resulted in the significantly augmented secretion of therapeutic MSC-derived EVs and their therapeutic contents (miRNAs and cytokines) from MSCs compared to conventional 2D cultures [7]. 

In the present study, we hypothesized that a clinical-scale EV product using a 3D micropatterned well system would enhance the wound healing process. To verify this, we developed an EV-bioprocessing platform designed using a cell non-adhesive microwell-patterned array for the scalable production of human Wharton’s jelly (WJ)-MSC-derived EVs in serum-free media. The effects of clinical-scale EV products on wound healing were tested in two different wound models: subcutaneous injection of EVs in a conventional full-thickness rat model and topical application of EVs using a sterile re-absorbable gelatin sponge in a chamber mouse model that was developed to prevent the contraction of wound areas. In addition, we performed in vitro and in vivo mechanistic studies using multiple cell lines involved in the wound healing process. 

## 2. Results

### 2.1. EV Characterisation

**Figure 1:**
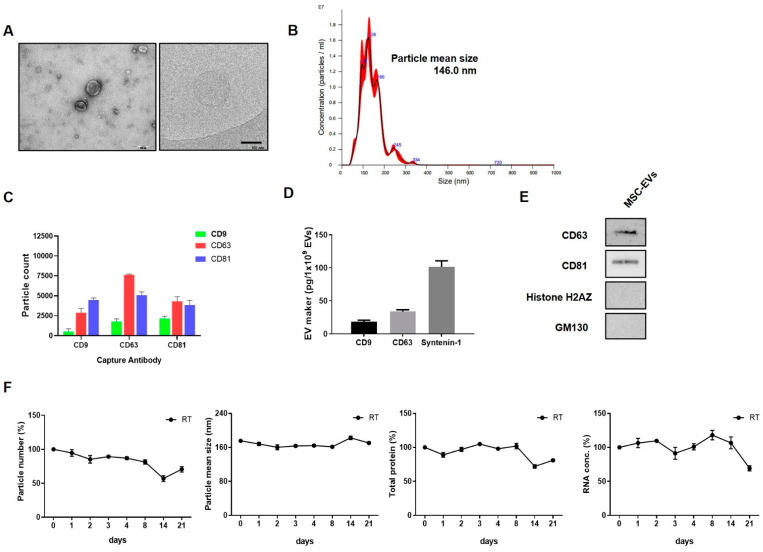
Characterization of extracellular vesicles (EVs) obtained from 3D culture system. (**A**) EVs imaged using electron microscopy (TEM and Cryo-EM); (**B**) histogram representing the size distributions and concentrations of the EVs using NanoSight. (**C**) The tetraspanins, including CD9, CD63, CD81, using Exoview analysis; and (**D**,**E**) EV positive markers, including CD63, CD81, syntenin-1 (enzyme-linked immunosorbent assay, ELISA) and EV negative markers, including histone H2A.Z and GM130 (Western blot). (**F**) Change in EVs according to storage period at RT.

The amount of EVs obtained from 3D culture system was estimated to be approximately 8155.28 EVs per cell. The EVs had a typical round shape as seen on electron microscopy (TEM and Cryo-EM) (Figure 1A), and the mean particle diameter was 146.0 nm (Figure 1B). We investigated the expression of CD9, CD63 and CD81 using the Exoview Tetraspanin kit. EVs were primarily captured by antibodies against each tetraspanin, and then fluorescently labeled by detection antibodies for the three tetraspanins. It was demonstrated that the subpopulation of CD63+ was higher than CD9+ or CD81+ (Figure 1C). The presence of EV-specific positive markers (CD63, CD81 and Syntenin-1) further confirmed the identity as EVs (Figure 1D). The particle/protein ratio was 6.5 × 10^8^ particles/μg. Specific contaminating proteins, including histone H2A.Z, GM130, and antibiotics, were identified by Western blot or ELISA. Antibiotics GM130 and histone H2A.Z were not detected (Figure 1E). The characteristics of EVs and their cargo contents did not change at room temperature after 1 week (Figure 1F). 

### 2.2. MSC-EVs Induce Re-Epithelialization in Both Types of Wound Models

**Figure 2:**
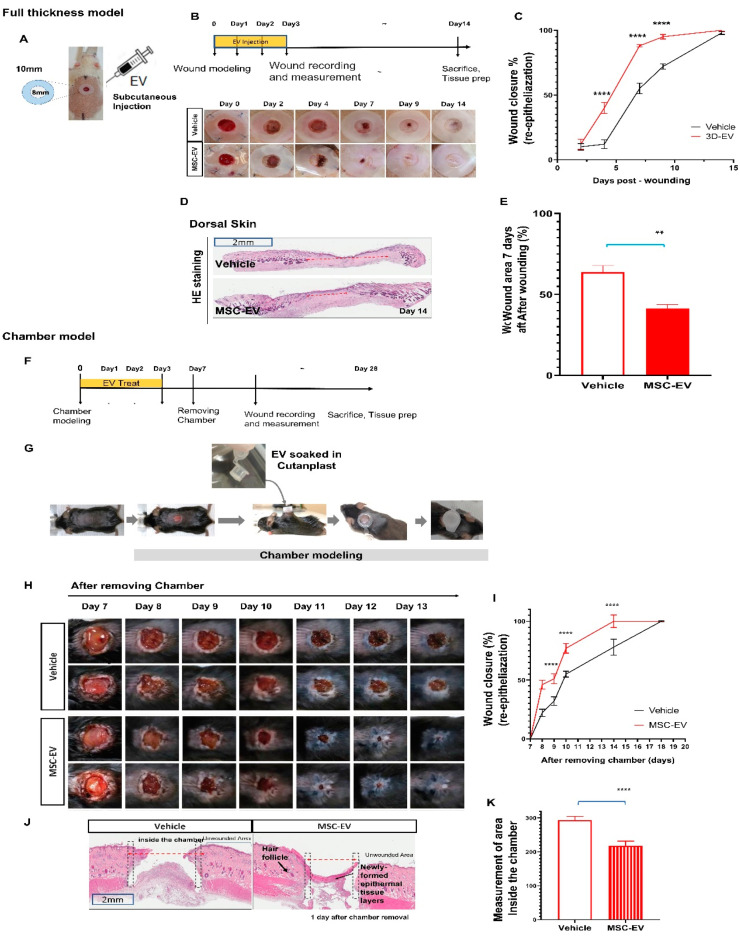
Evaluation of cutaneous wound healing in a conventional full-thickness rat wound model (**upper** panel) and a chamber mouse wound model (**lower** panel). (**A**) Schematic diagram of a full-thickness wound healing model (**B**) Representative image of a wound over 14 d. (**C**) Rate of wound closure (%, re-epithelialization) measured via histo-morphometric analysis of tissue sections. (**D**,**E**) Photomicrographs of hematoxylin and eosin (H&E)-stained histologic sections of wounded skin (Scanscope image, USA) 14 d post-wounding. Graph of measurements in panel (**E**). (**F**) Schematic diagram of the time schedule of the wound chamber model. (**G**) Surgical processes for the mouse skin excisional wound model. (**H**) Workflow for evaluating the mouse skin chamber wound model. (**I**) Increase in both wound closure percentage and the ratio of the area of granulation tissue to the wound field was observed in EV-treated mice. (**J**) Photo data of wound healing 1 d after chamber removal. (**K**) Semiquantitative evaluation of the gap width. Measurement of wound area percentage in the chamber. Thickness of the newly formed epidermis and measurement of the wound area inside the chamber. The number of animals used at each time point was six. Data represent the mean ± standard deviation (SD). **** *p* < 0.0001, ** *p* < 0.01.

To investigate the efficacy and mechanism of MSC-EVs in a full-thickness rat wound model, rats were induced into a full-thickness wound model, and 2 × 10^8^ EVs/rat were injected subcutaneously for 3 d (Figure 2A). Wound closure in the MSC-EVs group was higher than that in the PBS-treated group (Figure 2B,C). In addition, 14 d after wound induction, the contractility and repair ability of the wound center were measured, and the percentage of re-epithelialization was analyzed for wound repair capacity (Figure 2D,E). MSC-EV treatment significantly increased re-epithelization (Figure 2D) and reduced the size of the wound area compared with the controls (Figure 2E). 

In addition, we tested the effects of EVs in a mouse chamber wound model because, unlike in humans, rodent skin wounds contract soon after wound formation (Figure 2F). In the chamber model, topical application of EVs using a sterile re-absorbable gelatin sponge (Cutanplast) in the chamber mouse model induced wound closure (Figure 2G,H) and improved re-epithelialization and granulation tissue in the chamber (Figure 2I,J). MSC-EVs induced a significant reduction in the size of the wound areas (%) in the chamber, strengthened the newly formed epidermal layer, and promoted the production of granulation tissue in the chamber. 

### 2.3. MSC-EVs Accelerate Wound Healing by Promoting the Migration of Keratinocytes

**Figure 3:**
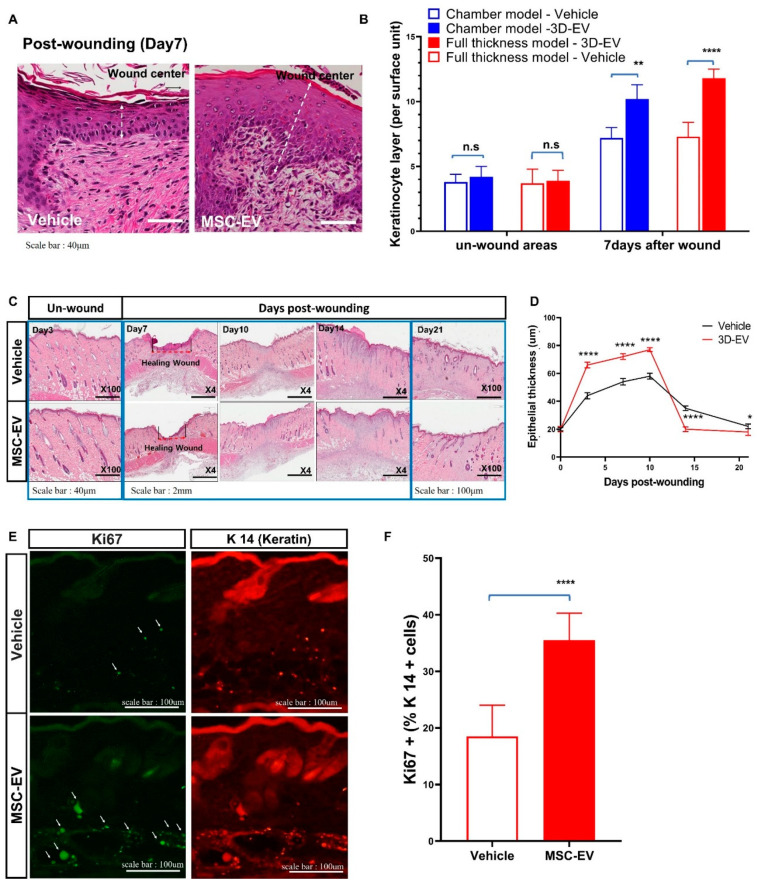
EVs promote the proliferation and migration of keratinocytes during the initial epidermal hypertrophy process. (**A**,**B**) Mesenchymal stem cell (MSC)-EVs accelerate wound healing with individually migrating keratinocytes in a mouse chamber model. Scale bar: 40 μm. (**C**) H&E-stained histopathological whole-slide images of full-thickness wounds at 3, 7, 10, 14, and 21 d post-wounding. (Unwounded, day 3, magnification, 100×, scale bar, 100 μm), (days post-wounding, from day 7 to 14, magnification, 4×, scale bar, 2 mm), (days post-wounding, from day 21, magnification, 100×, scale bar, 100 μm). (**D**) Serial changes in epidermal thickness were measured and compared between the EV and control groups. (**E**) Immunohistochemical image of the epidermis and (**F**) measurement of epidermal thickness 7 d after wound induction. White arrows indicate Ki67+/keratinocyte cells. Scale bar: 100 m. Data represent the mean ± SD. ns: *p* > 0.05, not significant, **** *p* < 0.0001, ** *p* < 0.01, and * *p* < 0.05.

MSC-EVs stimulated epithelial regeneration in both wound models. MSC-EVs promoted hypertrophy of the epithelial cell layer after 3 d of treatment with EVs (Figure 3A,B). Immunohistochemical examination 7 d after wounding showed that the number of keratinocytes was increased in the epithelial cell layer, suggesting that MSC-EVs promote the proliferation of keratinocytes for re-epithelization (Figure 3E,F). Interestingly, the epithelial cell layers returned to normal thickness after 2 weeks of MSC-EV treatment (Figure 3C,D), suggesting that EV-mediated regeneration of the epidermis occurs mainly during the initial phase of wound healing and the remodeling of the scar tissue maturation phase. In a study of wound tissue treated with MSC-EV, it was observed that the skin tissue underwent stabilization and thinning during the maturation stage. Immunohistochemistry for keratin 14 (a marker of keratinocyte cells) and Ki-67 (a marker of proliferating cells) showed that MSC-EVs stimulated the proliferation and migration of keratinocytes (Figure 3E,F).

### 2.4. MSC-EVs Promote the Migration of Mature Fibroblasts into the Granulation Tissue

**Figure 4:**
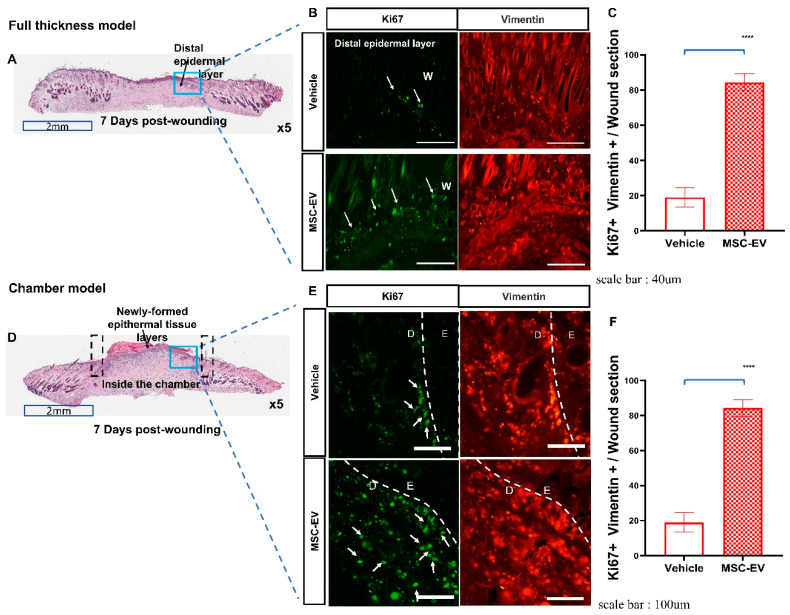
EVs promote fibroblast cell proliferation and migration. (**A**,**D**) Representative cross-sectional images of H&E-stained mouse dorsal skin with epidermal structures of MSC-EV-treated wounds. Areas marked with black lines represent the Chambers, blue boxes indicate the newly created granulation tissue, and downward arrows indicate the newly formed epidermis. Scale bar: 2 mm. (**B**,**E**) Ki-67-positive fibroblast cells (GFP-TRITC merged cells) in typical cross-sections of distal epidermal layer of the vehicle group or MSC-EV-treated group. (Panels (**A**,**D**): magnification, 5×, scale bar: 2 mm), (Panel (**B**): Full thickness wound model, scale bar: 40 μm), (Panel (**E**): Chamber model, scale bar: 100 μm). D: Dermal tissue; E: Epidermal tissue; W: wound. (**C**,**F**) Quantification of the percentage of fibroblasts expressing the Ki-67 antigen/wound areas. Data represent the mean ± SD. **** *p* < 0.0001.

MSC-EV therapy stimulated the proliferation of fibroblasts to promote the maturation of granulation tissue in both the full-thickness and chamber wound models (Figure 4). Treatment with MSC-EVs increased the number of proliferating fibroblasts that were positive for both Ki67 and vimentin (a marker of fibroblast cells) in immunological staining (Figure 4B,E). In addition, the migration of proliferating fibroblasts to the granulation tissue was increased after treatment with MSC-EVs, from subcutaneous areas in the chamber model and from the non-injured regions in the full-thickness model (Figure 4A,D). 

### 2.5. MSC-EVs Promote the Formation of New Blood Vessels in the Wound Area 

**Figure 5:**
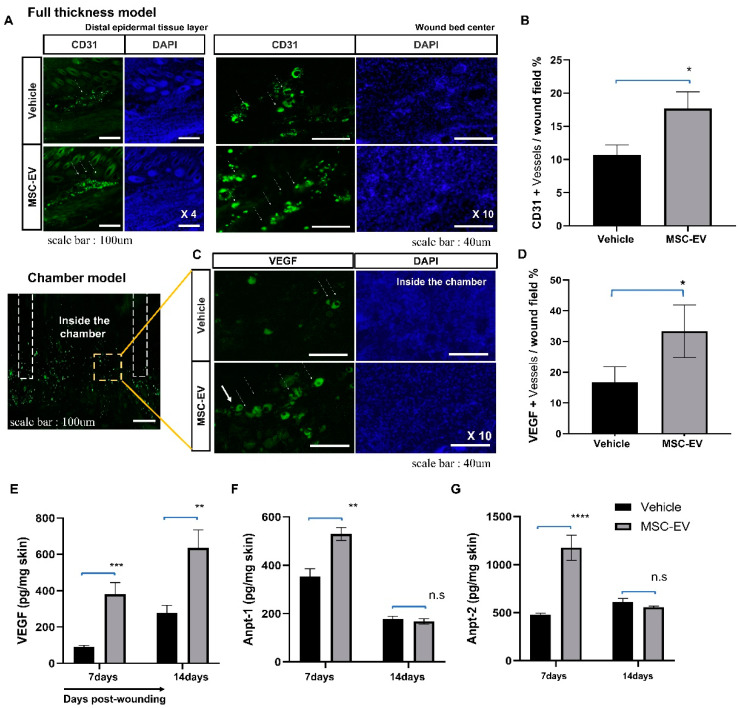
Effects of EVs on angiogenesis in the chamber wound model. (**A**) Immunohistochemical staining for CD31 (a marker of vascular structure) in the dermis and neovascularization sites at the center of the wound. Arrow heads indicate CD31 + vessels. Left panel, scale bar: 100 μm; Right panel, scale bar: 40 μm (**B**) Quantification of CD31^+^ cells and vessels per 20× field in the wound bed at the center of wounds treated with the vehicle or MSC-EVs collected on day 7 post-wounding. (**C**) On day 14, vascular endothelial growth factor (VEGF)-positive blood vessels were observed in MSC-EV-injected wounds. Arrow heads indicate VEGF + vessels. Scale bar: 40 μm (**D**) Quantification of VEGF^+^ cells and vessels per 20× field in the wound bed at the center of wounds treated with the vehicle or MSC-EVs collected on day 14 post-wounding. (**E**–**G**) ELISA analyses of angiogenesis protein expression in MSC-EV-treated wound and vehicle groups. Each sample was assessed in duplicate, and the analysis was conducted thrice independently. Error bars indicate the mean ± SD. *p*-values were calculated using an unpaired Student’s *t*-test. **** *p* < 0.0001, *** *p* < 0.001, ** *p* < 0.01, and * *p* < 0.05; ns: *p* > 0.05, not significant.

Immunohistochemical staining for CD31 (a marker of vascular structure) showed that MSC-EVs enhanced the vascular structure in both the epithelial cell layer and the wound center region during the wound healing process (Figure 5A). Similarly, immunohistochemical staining for a vascular endothelial growth factor (VEGF, a blood vessel marker) showed that MSC-EVs promoted angiogenesis (Figure 5C). We also measured the tissue levels of pro-angiogenic growth factors and found that VEGF, angiopoietin (Anpt)-1, and Anpt-2 levels were significantly increased in tissue lysates obtained from the dorsal wound area in the EV group compared to those in the control group (Figure 5E–G).

### 2.6. In Vitro Assay for MSC-EV Effects on Four Major Cell Types, Fibroblasts, Keratocytes, Endothelial Cells, and Inflammatory Cells

**Figure 6:**
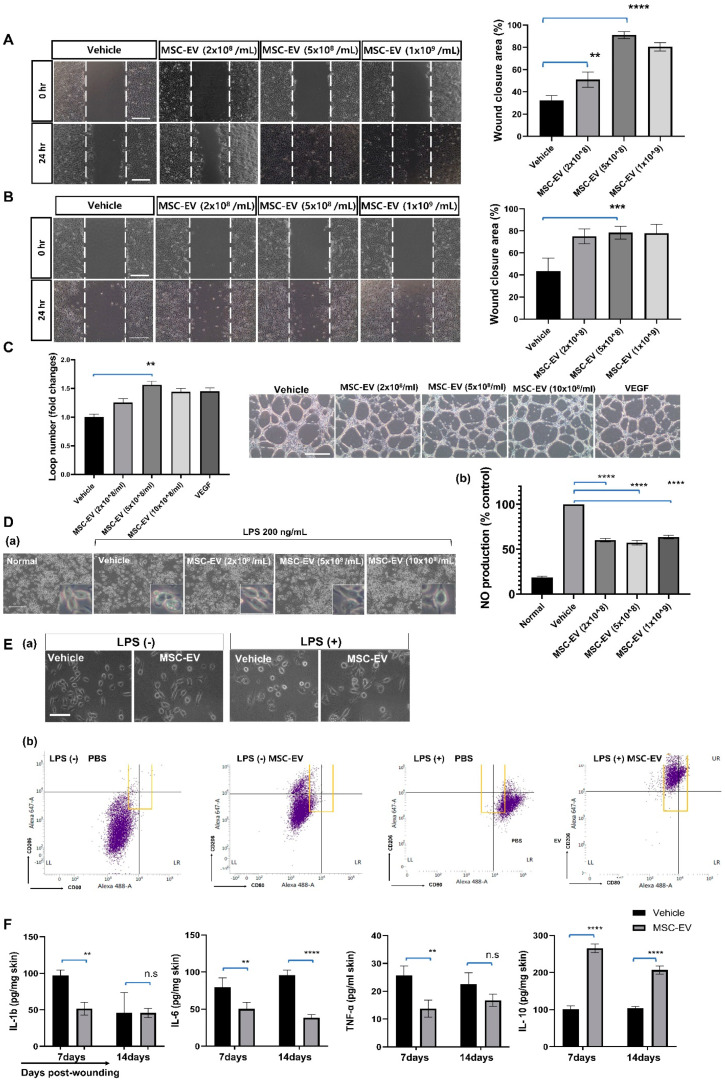
In vitro assay for MSC-EV effects on four major cell types involved in wound healing: keratinocytes, fibroblasts, endothelial cells, and inflammatory cells. (**A**,**B**) Scratch wound healing, tube formation, and release of VEGF in vitro assays. Scratched HaCaT (**A**) and NIH-3T3 (**B**) cell monolayer treated with MSC-EVs and imaged after 24 h. Wound closure area was determined via ImageJ software analysis. Data are presented as the means of three independent measurements. Magnification: ×10. *** *p* < 0.001, ** *p* < 0.01, and ns: *p* > 0.05, not significant. compared to the vehicle group. Scale bar 400 μm. (**C**) Human umbilical vein endothelial cells (HUVECs) cultured in a Matrigel-coated 96-well plate were incubated with different doses of EVs for 24 h. Representative images of HUVEC tube formation are shown, and endothelial tube formation in each group was quantified. Scale bar 500 μm. (**D**) RAW264.7 cells were pretreated with various concentrations (2, 5, and 10 × 10^8^/mL) of EVs with lipopolysaccharide (LPS; 200 ng/mL) for 24 h. Cell morphology was visualized via optical microscopy (Scale bar 100 μm, Panel (**a**)). Data are expressed as the mean ± standard error of the mean (SEM) of three independent experiments (Panel (**b**)). Percentage viability of RAW264.7 cells untreated (normal group), treated with only 200 ng/mL LPS (positive control), and treated with various particle numbers of MSC-EVs in the presence of 200 ng/mL LPS for 24 h. Data are represented as the mean ± SD of three independent experiments. Scale bar 100 μm (**E**) Macrophage polarization. To examine the effects of MSC-EVs on macrophage differentiation, we treated RAW264.7 cells in the presence or absence of LPS with traditional M1 and M2 macrophage-differentiated cytokines followed by flow cytometry analysis with CD80 and CD206 surface markers. MSC-EVs dominantly promoted macrophage cells toward CD206^+^ (M2 marker), not CD80^+^ (M1 marker) cell differentiation on RAW264.7 cells (**E**). Scale bar 40 μm. Panel (**a**): Microscopic images depicting the morphology of cells at different stages of the polarization protocol. Panel (**b**): M2 macrophages stimulated with increasing concentrations of EVs in 200 ng/mL LPS. (**F**) Quantitative analysis of pro-inflammatory (IL-6, IL-1b, and tumor necrosis factor-α) and anti-inflammatory cytokines (IL-10) in mouse skin wounds via ELISA. Data are represented as the mean ± SD. The results are representative of three experiments. Student’s *t*-test was used for analysis. **** *p* < 0.0001, *** *p* < 0.001, ** *p* < 0.01, and ns: *p* > 0.05, not significant.

We performed in vitro studies to investigate the mechanisms of MSC-EVs using multiple cell lines involved in the wound healing process: keratinocytes (HaCaT), fibroblasts (NIH-3T3), endothelial cells (HUVECs), and inflammatory cells (RAW264.7). For both NIH-3T3 and HaCaT cells, cell motility was assessed using a scratch wound model. Various MSC-EVs (2, 5, and 10 × 10^8^ EVs) were administered for 24 h (Figure 6A,B). MSC-EVs promoted the proliferation of both keratinocytes and fibroblasts, although the maximal effective dose was lower in fibroblasts than in keratinocytes. The tube formation assay using HUVECs showed a dose-dependent increase in angiogenesis (Figure 6C). Lastly, inflammation-induced macrophage RAW264.7 cells were tested using the Griess reagent for NO production (Figure 6D). Treatment with MSC-EVs promoted the polarization of M2-type macrophages (Figure 6E). In addition, compared to the control group, the levels of inflammatory cytokines were significantly decreased, but the levels of anti-inflammatory cytokines (IL-10) were increased in the EV group (Figure 6F).

## 3. Discussion

This study is the first to show that clinical-scale EV therapeutics are feasible using a micro-patterned well system and can improve the wound healing process. In this study, the effects of EV treatment were tested in different wound injury models under different treatment modes, which showed consistent findings. The mechanisms of action of MSC-EVs were assessed using both in vivo and in vitro models. The therapeutic potential of EVs can contribute to multiple stages of wound healing, such as cell proliferation and differentiation, inflammation, angiogenesis, and extracellular matrix remodeling. Specifically, our clinical-scale EV therapeutics could effectively induce the proliferation and migration of endothelial cells, keratinocytes, and fibroblasts to improve angiogenesis and re-epithelialization and regulate inflammatory cells in rodent wound models. 

To date, multiple studies have investigated the effects of stem cell-derived EVs in wound models [8,9,10,11,12,13,14,15,16,17,18]. MSC-EV therapies obtained from various MSC sources, such as bone marrow, adipose tissue, and umbilical cord, have been used to improve recovery in various wound models. However, the development of MSC-EV therapeutics faces several hurdles, including establishing a consistent, scalable cell source and developing robust GMP-compliant upstream and downstream manufacturing processes [19]. MSCs undergo senescence, and their intrinsic ability to secrete EVs significantly declines in conventional 2D cultures; therefore, MSC-EV preparations may differ in their therapeutic potential. In addition, according to the US FDA conversion guideline documents for industry estimating the maximum safe starting dose in adult healthy volunteers (July 2005), one patient in clinical testing requires more than 100 times higher doses than those of one mouse or rat. Low output limits of EV preparations obtained from the conventional 2D culture of MSCs limit the clinical application of EVs. EVs obtained under 3D cultures, such as micro-patterned well systems, as shown in the present study, hollow fiber bioreactor-based 3D culture systems, and 3D scaffolds cultures, exhibited enhanced EV yield and a heightened damage-repair ability [20,21]. Therefore, for effective clinical-scale production of therapeutic EVs, large batches of MSCs are needed, which significantly affects the labor, time, and cost of production. In this study, we established a cell bank, used the 3D culture method, and the combination of filter and TFF system, as it allowed the large-scale production of EVs (the yield of EVs is more than 10–20 fold that of conventional 2D culture) without the use of serum. Compared to conventional stem cell-based therapeutics, our EV therapy has potential benefits in terms of cost-effectiveness when WJ-MSCs are cultured in a 3D micropatterned well system and isolated using a TFF system (Supplementary Figure S2). More importantly, our scalable 3D-bioprocessing EV production method reduced the donor/batch variation. Lastly, our small RNA sequencing data revealed that MSC-EVs containing miRNAs played important roles in angiogenesis, cytoprotection, immune modulation, and rejuvenation, and miRNAs, such as miR-21-3p, miR-125a, and miR-126-3p, were involved in the wound healing process after treatment with MSC-EVs (Supplementary Figure S3) [8,9,10,14,22,23]. MSC-EVs treatment has been found to promote wound healing by increasing the expression of VEGF-A, Wnt, and PI3K/AKT in fibroblast and keratinocyte cells. These findings suggest that EV-contained miRNA and cargo play a key role in wound healing by regulating specific signaling pathways, but more research is needed to fully understand the mechanism and potential therapeutic applications of MSC-EVs in wound healing (Supplementary Figures S3 and S4) [8].

In this study, the effects of MSC-EV treatment were tested in different species (mouse and rat) and wound models (mild [traditional full-thickness model] and severe [chamber model]), which showed consistent therapeutic benefits. The chamber model prevents the migration of keratinocytes into the wound and the closure of the wound via contraction [24]. It facilitates the de novo generation of epithelial tissues from the surface of the skin ulcers. Our results suggest that the application of EVs stimulates wound-resident stem cells to promote the wound-healing process; however, further studies are required to evaluate the de novo generation of epithelial tissues from wounded tissues [24].

Wound healing is classically divided into four stages: hemostasis, inflammation, proliferation, and remodeling. Each stage is characterized by key molecular and cellular events and is coordinated by a host of secreted factors that are recognized and released by the cells of the wounding response [25]. As various cellular components are involved at different stages of the wound healing process, we performed an in vitro assay to determine EV effects on four major cell types: fibroblasts, keratocytes, endothelial cells, and inflammatory cells. Depending on the severity and chronology (time interval from the onset of wound injury) of the wound and the presence of any comorbidities, such as infection and diabetes mellitus, in patients, one stage may be more prominent than others, and the target of treatment could be different among patients. For example, therapies with anti-inflammatory effects are needed in the inflammatory phase, the first phase after the cutaneous wound, while enhancing angiogenesis can be an important strategy in patients with diabetes mellitus. Proliferation and remodeling are important targets for the treatment of chronic deep wounds. The in vitro assay can aid in assessing the targets for different wound healing treatments. The results of this study showed that MSC-EV therapeutics exert their effects in most phases of wound healing. 

This study has several limitations. First, the molecular action mechanisms of MSC-EVs could not be investigated. Of the cargo in exosomes, miRNAs are of prime importance in mediating the therapeutic effects on wound healing [8,9,10,11]. Molecular pathways of EV-miRNAs involved in wound healing are under investigation. In addition, we studied the effects of MSC-EVs in healthy young mice and rats. Cutaneous wounds are difficult to heal in older patients and those with comorbidities, especially diabetes mellitus. We are currently investigating the effects of MSC-EVs in diabetic wound animal models. Lastly, further in vivo studies are needed to determine the dose-responsiveness and optimal dose of EVs based on the specific phase of wound healing, as the optimal doses for angiogenesis and proliferation of keratinocytes and fibroblasts were different in our in vitro studies.

In conclusion, the present study demonstrated that our scalable 3D-bioprocessing production method is feasible for clinical-scale MSC-EV therapy. Moreover, our results showed that MSC-EVs promote wound healing in both mild and severe injuries via the regulation of various wound-healing phases. 

## 4. Materials and Methods

All studies involving human subjects were approved by the Institutional Review Board of Samsung Medical Center. WJ was provided to the healthy volunteers. All volunteers or their guardians provided written informed consent to participate in the study. All experimental animal procedures were approved by the Institutional Animal Care and Use Committee (Laboratory Animal Research Center, AAALAC International approved facility) of Samsung Medical Center.

### 4.1. Preparation of EV-Three-Dimensional (3D) Spheroid Cultures of WJ-MSCs

MSCs derived from human WJ of the umbilical cord (WJ-MSCs) were culture expanded at passage five with growth medium in a 5% CO_2_ incubator at 37 °C. WJ-MSCs were used at passage six to generate 3D spheroid cultures. WJ-MSCs were seeded into a micro-patterned well system (EZSPHERE; ReproCELL Inc., Tokyo, Japan), washed with phosphate-buffered saline (PBS), and trypsinized using TrypLE Express (GIBCO, NY, USA). After the WJ-MSCs were centrifuged, a fresh serum-free medium without heterologous proteins was added, and the cells were counted using a hemocytometer. After cell counting, 60 mL of the cell suspension was placed in a microarray containing approximately 69,000 microwells, each with a diameter and depth of 500 μm × 200 μm coated with 2-methacryloyloxyethyl phosphorylcholine polymer at a density of 400 cells/well. For the 3D spheroid culture of WJ-MSC, serum-free medium (α-minimal essential medium) was used, without any antibiotic. A 3D spheroidal cell aggregate was prepared by inducing spontaneous spheroidal cell aggregate formation while maintaining a static state by dispensing uniformly and culturing in a CO_2_ incubator at 37 °C for 4 d.

### 4.2. Isolation of EVs

EV isolation was performed in a biological safety cabinet. The culture medium was collected via gentle pipetting at the top of each well. To remove the cell debris and apoptotic bodies, 1800 mL of culture medium was centrifuged at 2500× *g* for 10 min, followed by filtration through a 0.22-μm membrane. The filtered medium was separated using a 300-kDa MWCO mPES hollow fiber MiniKros filter module (Spectrum Laboratories, Rancho Dominguez, CA, USA) on a commercially available KrosFlo KR2I tangential flow filtration (TFF) system (Spectrum Laboratories, Rancho Dominguez, CA, USA), which facilitates the large-scale processing of samples. EV-containing samples were recirculated into a filtration bottle. Small molecules, including free proteins, were passed through the membrane pores, eluted as a permeate, and collected. The collected solution was used as the secretome. EVs were maintained in circulation as retentate and concentrated in the bag. We conducted five volume exchanges of EVs with PBS, and EVs were subsequently concentrated to a final volume of 300 mL of recovery solution (PBS). The recovered solution was filtered through a 0.22-μm membrane. After harvesting the conditioned media, the EV isolation process was started immediately using the TFF procedure.

All processes were performed according to the guidelines on quality, non-clinical, and clinical assessment of EV therapy products of the Korean Food and Drug Administration (FDA, released December 2018) using good manufacturing practice (GMP)-compliant methods. Schematics of the processes of EV production, isolation, and quality control are shown in Supplementary Figure S1.

### 4.3. Characterization of EVs

Following the guidelines recommended by the International Society for Extracellular Vesicles (Minimal Information for Studies of Extracellular Vesicles 2018) and the Korean FDA, EVs isolated from the WJ-MSC culture medium were characterized in terms of their morphology, size distribution, surface markers, purity, potency markers, efficacy, stability, and safety [26]. 

See the Supplementary detailed methods for nanoparticle tracking analysis, Western blotting, transmission electron microscopy (TEM), enzyme-linked immunosorbent assay (ELISA), Exoview analysis, quantitative reverse transcription-polymerase chain reaction, and small RNA sequencing.

### 4.4. Two Animal Models of Cutaneous Wound

All animal experiments were approved by the Institutional Animal Care and Use Committee of Samsung Biomedical Research Institute and performed in accordance with the Institute of Laboratory Animal Resources guidelines. All animals were maintained in compliance with the relevant laws and institutional guidelines of the Laboratory Animal Research Center (AAALAC International-approved facility) at Samsung Medical Center. 

#### 4.4.1. Conventional Full-Thickness Skin Wound Rat Model

A conventional full-thickness cutaneous wound model was used in this study. Briefly, excisional wounds were created using an 8 mm diameter punch (Acuderm, Inc., Ft. Lauderdale, FL, USA) on the shaved dorsal skin under ketamine (100 mg/kg) and xylazine hydrochloride (5 mg/kg) anesthesia. Silicone splints were fixed around the excised wound. EVs were injected subcutaneously at four different points around the wounds, while an equal volume of PBS was injected subcutaneously in the same position in the control group rats. Based on the results of our preliminary experiments, a dose of 2 × 10^8^ EVs/rat was selected for further experiments using the rat model. 

#### 4.4.2. Mouse Chamber Wound Model

Unlike human skin, rodent skin has panniculus carnosus, a thin layer of muscle attached to the subcutaneous tissue that acts as a contractile force for wound closure. Therefore, in the full-thickness rat model, it was difficult to measure the regeneration and recovery mechanisms of skin epithelial cells because of rapid wound healing by contraction. Therefore, we tested the effects of EVs in a mouse chamber model [24,27].

We surgically removed the skin from the back of the mice to generate an ulcer and isolated the resulting wound from the surrounding skin using a skin chamber sutured to the deep fascia. A chamber-made EP tube was placed inside the skin layer and fixed to the skin layer by a simple suture. Since mice are half as small as rats based on their body surface area, a dose of 1 × 10^8^ EVs/mouse was selected for the mouse model and applied for 3 d after a full-thickness excision wound. Cutanplast was moistened with EVs and placed inside the chamber. To prevent inflammation in the chamber, antibiotics (Baytril) were injected for 2 weeks after surgery.

### 4.5. Measurement of Wound Contraction

Measurements of wound contraction and wound closure were performed using surgical calipers, and the wound areas were quantified using Aperio Image Scope V 12 software. Wounds were photographed on days 0, 1, 3, 5, 7, 10, 14, and 21 post-wounding, and wound size was determined using the ImageJ software (National Institutes of Health, Bethesda, MD, USA) to measure the wound area. The percentage of wound closure was calculated using the following equation:

$$Wound\;closure=\frac{Initial\;wound\;size-Specific\;day\;wound\;size}{Initial\;wound\;size}\times 100$$

Using histological samples, the general linear model for the determination of time versus wound closure (re-epithelialization) and granulation tissue formation for each treatment was evaluated. Wound contraction was calculated as a percentage of the original wound size, taken as 100% of each animal in the group using the equation given above. The percentage of wound area was calculated using the following formula:

$$Wound\;area\;\left(\%\right)=\frac{Area\;at\;biopsy}{Area\;on\;incision\;day}\times 100$$

### 4.6. In Vivo Efficacy Test and Mechanism Study

#### 4.6.1. Histological Analysis

Skin tissue samples were fixed in 4% paraformaldehyde for 24 h and underwent dehydration with graded ethanol. The samples were then embedded in an optimal cutting temperature compound and cut into 10–30-μm thick sections. Hematoxylin and eosin (H&E) staining was performed using commercial staining kits (H&E Staining Kit (ab245880), Abcam, Cambridge, UK)), according to the manufacturer’s instructions. Images were captured using a microscope (ScanScope image, USA).

#### 4.6.2. Immunohistochemistry

After 15 d of induction of wound models, the effect of MSC-EVs was compared with that of the control (basal medium) by immunostaining with Ki-67 (a cell proliferation marker) and vimentin (a fibroblast marker), according to the manufacturer’s instructions. Dorsal skin tissues were fixed in 4% paraformaldehyde and blocked with 10% normal goat serum. Dorsal skin was incubated overnight at 4 °C with rabbit anti-Ki-67 (1:50; Abcam, UK) and goat anti-vimentin (1:500; Abcam, UK) antibodies. The cells were then washed with PBS and incubated with secondary DyLight-labeled anti-goat IgG (1:200, 594 nm; Abcam, UK) and DyLight-labeled anti-rabbit IgG (1:200, 488 nm; Vector Laboratories, Burlingame, CA, USA) antibodies. Samples were imaged using a fluorescence microscope (EVOS; Advanced Microscopy Group, Bothell, WA, USA), and positively stained cells were quantified using ImageJ software.

#### 4.6.3. Measurement of Cytokine Levels via ELISA

ELISA was performed using commercial kits according to the manufacturer’s instructions. The following ELISA kits were used: tumor necrosis factor-α (MBS140025, MyBioSource, San Diego, CA, USA), Ang-1 (MBS2601637, MyBioSource, San Diego, CA, USA), Ang-2 (MBS8420366, MyBioSource, San Diego, CA, USA), interleukin (IL)-10 (MBS140013, MyBioSource, San Diego, CA, USA), IL-6 (MBS 824703, MyBioSource, San Diego, CA, USA) and IL-beta (MBS 175967, MyBioSource, San Diego, CA, USA). All kits included standard proteins; therefore, the amount of protein and EV counts were determined based on the standard curve from each kit.

### 4.7. In Vitro Efficacy Test and Mechanism Study

#### 4.7.1. Measurement of Nitric Oxide Production in RAW264.7 Cells

The level of NO was determined by measuring the quantity of nitrite in the supernatant using the Griess reaction. Macrophage RAW264.7 cells (1.0 × 10^5^) were seeded into a 24-well plate and treated with lipopolysaccharide (LPS; 100 ng/mL) for 24 h. To measure the amount of NO produced, 50 μL of conditioned medium was mixed with an equal volume of Griess reagent (Sigma, Saint Louis, MO, USA) and incubated for 15 min at room temperature. Absorbance was measured at 540 nm using a microplate reader, and the absorbance versus sodium nitrite concentration plot was constructed. 

#### 4.7.2. Fibroblast Wound Healing Assay in NIH-3T3 Cells

NIH-3T3 cells were seeded at 1.8 × 10^5^/well into a 12-well plate. The wells were then scratched longitudinally using a yellow tip. After washing twice with high glucose media, cultures were treated with the same medium containing 5 μg/mL mitomycin C (Sigma, Saint Louis, MO) with or without MSC-EVs (2, 5, and 10 × 10^8^ /mL). Cell migration was assayed 24 h after MSC-EV treatment using optical microscopy. Wound areas were measured using the ImageJ software, and the percentage of cell motility was calculated using the following equation: ([Area at 0 h − Area at 12 h]/Area at 0 h) × 100. 

#### 4.7.3. Keratinocyte Wound Healing Assay in HaCaT Cells

HaCaT cells were seeded at 2.2 × 10^5^/well into a 12-well plate. The experimental procedure was the same way as the one used in the NIH-3T3 fibroblast wound-healing assay.

#### 4.7.4. Angiogenesis Assay in Human Umbilical Vein Endothelial Cells 

In vitro capillary network formation was determined using a tube formation assay on Matrigel (354248; Corning, Glendale, AZ, USA). Human umbilical vein endothelial cells (HUVECs) (1.5 × 10^4^ cells/mL) were seeded onto Matrigel-coated wells of a 96-well plate and cultured in 1% fetal bovine serum-supplemented Dulbecco’s Modified Eagle’s medium (10567014; Gibco, Waltham, MA USA) in the presence of 5 × 10^8^/mL MSC-EVs or PBS. Tube formation was observed using an inverted microscope (Leica DMi8, Wetzlar, Germany). The number of network structures was quantified by randomly selecting five fields per well using ImageJ software.

### 4.8. Statistical Analyses

Statistical analyses were conducted using the SPSS program (SPSS Statistics Version 24.0, IBM Corp, Armonk, NY, USA) and GraphPad Prism 9 software (GraphPad Software, San Diego, CA, USA). The normality of the data was evaluated using the D’Agostino–Pearson test. One- and two-way analyses of variance with Tukey’s multiple comparison tests were used to analyze the three groups. Student’s *t*-test and Wilcoxon–Mann–Whitney test were used for paired and unpaired analyses of the two groups. Statistical analysis results are indicated in the figure legends. Results are expressed as the mean ± standard error. Statistical significance was defined as *p* < 0.05.

## Data Availability

Not applicable.

## Supplementary Materials

The following supporting information can be downloaded at: https://www.mdpi.com/article/10.3390/ijms24054273/s1.

## References

[1] Doi H., Kitajima Y., Luo L., Yan C., Tateishi S., Ono Y., Urata Y., Goto S., Mori R., Masuzaki H. (2016). Potency of umbilical cord blood- and Wharton’s jelly-derived mesenchymal stem cells for scarless wound healing. Sci. Rep..

[2] Maxson S., Lopez E.A., Yoo D., Danilkovitch-Miagkova A., Leroux M.A. (2012). Concise review: Role of mesenchymal stem cells in wound repair. Stem Cells Transl. Med..

[3] Badiavas E.V., Falanga V. (2003). Treatment of chronic wounds with bone marrow-derived cells. Arch Derm..

[4] Vojtassak J., Danisovic L., Kubes M., Bakos D., Jarabek L., Ulicna M., Blasko M. (2006). Autologous biograft and mesenchymal stem cells in treatment of the diabetic foot. Neuro Endocrinol. Lett..

[5] Squillaro T., Peluso G., Galderisi U. (2016). Clinical Trials With Mesenchymal Stem Cells: An Update. Cell Transpl..

[6] Zaher W., Harkness L., Jafari A., Kassem M. (2014). An update of human mesenchymal stem cell biology and their clinical uses. Arch Toxicol..

[7] Cha J.M., Shin E.K., Sung J.H., Moon G.J., Kim E.H., Cho Y.H., Park H.D., Bae H., Kim J., Bang O.Y. (2018). Efficient scalable production of therapeutic microvesicles derived from human mesenchymal stem cells. Sci. Rep..

[8] Hu Y., Rao S.S., Wang Z.X., Cao J., Tan Y.J., Luo J., Li H.M., Zhang W.S., Chen C.Y., Xie H. (2018). Exosomes from human umbilical cord blood accelerate cutaneous wound healing through miR-21-3p-mediated promotion of angiogenesis and fibroblast function. Theranostics.

[9] Lv Q., Deng J., Chen Y., Wang Y., Liu B., Liu J. (2020). Engineered Human Adipose Stem-Cell-Derived Exosomes Loaded with miR-21-5p to Promote Diabetic Cutaneous Wound Healing. Mol. Pharm..

[10] Tao S.C., Guo S.C., Li M., Ke Q.F., Guo Y.P., Zhang C.Q. (2017). Chitosan Wound Dressings Incorporating Exosomes Derived from MicroRNA-126-Overexpressing Synovium Mesenchymal Stem Cells Provide Sustained Release of Exosomes and Heal Full-Thickness Skin Defects in a Diabetic Rat Model. Stem Cells Transl. Med..

[11] Gao S., Chen T., Hao Y., Zhang F., Tang X., Wang D., Wei Z., Qi J. (2020). Exosomal miR-135a derived from human amnion mesenchymal stem cells promotes cutaneous wound healing in rats and fibroblast migration by directly inhibiting LATS2 expression. Stem Cell Res. Ther..

[12] Yu M., Liu W., Li J., Lu J., Lu H., Jia W., Liu F. (2020). Exosomes derived from atorvastatin-pretreated MSC accelerate diabetic wound repair by enhancing angiogenesis via AKT/eNOS pathway. Stem Cell Res. Ther..

[13] He X., Dong Z., Cao Y., Wang H., Liu S., Liao L., Jin Y., Yuan L., Li B. (2019). MSC-Derived Exosome Promotes M2 Polarization and Enhances Cutaneous Wound Healing. Stem Cells Int..

[14] Choi J.S., Cho W.L., Choi Y.J., Kim J.D., Park H.A., Kim S.Y., Park J.H., Jo D.G., Cho Y.W. (2019). Functional recovery in photo-damaged human dermal fibroblasts by human adipose-derived stem cell extracellular vesicles. J. Extracell Vesicles.

[15] McBride J.D., Rodriguez-Menocal L., Guzman W., Candanedo A., Garcia-Contreras M., Badiavas E.V. (2017). Bone Marrow Mesenchymal Stem Cell-Derived CD63(+) Exosomes Transport Wnt3a Exteriorly and Enhance Dermal Fibroblast Proliferation, Migration, and Angiogenesis In Vitro. Stem Cells Dev..

[16] Zhang B., Wang M., Gong A., Zhang X., Wu X., Zhu Y., Shi H., Wu L., Zhu W., Qian H. (2015). HucMSC-Exosome Mediated-Wnt4 Signaling Is Required for Cutaneous Wound Healing. Stem Cells.

[17] Liu J., Yan Z., Yang F., Huang Y., Yu Y., Zhou L., Sun Z., Cui D., Yan Y. (2020). Exosomes Derived from Human Umbilical Cord Mesenchymal Stem Cells Accelerate Cutaneous Wound Healing by Enhancing Angiogenesis through Delivering Angiopoietin-2. Stem Cell Rev. Rep..

[18] Zhao G., Liu F., Liu Z., Zuo K., Wang B., Zhang Y., Han X., Lian A., Wang Y., Liu M. (2020). MSC-derived exosomes attenuate cell death through suppressing AIF nucleus translocation and enhance cutaneous wound healing. Stem Cell Res. Ther..

[19] Johnson J., Shojaee M., Mitchell Crow J., Khanabdali R. (2021). From Mesenchymal Stromal Cells to Engineered Extracellular Vesicles: A New Therapeutic Paradigm. Front. Cell Dev. Biol..

[20] Luo Y., Li Z., Wang X., Wang J., Duan X., Li R., Peng Y., Ye Q., He Y. (2022). Characteristics of culture-condition stimulated exosomes or their loaded hydrogels in comparison with other extracellular vesicles or MSC lysates. Front. Bioeng. Biotechnol..

[21] Lv D., Hu Z., Lu L., Lu H., Xu X. (2017). Three-dimensional cell culture: A powerful tool in tumor research and drug discovery. Oncol. Lett..

[22] Fang S., Xu C., Zhang Y., Xue C., Yang C., Bi H., Qian X., Wu M., Ji K., Zhao Y. (2016). Umbilical Cord-Derived Mesenchymal Stem Cell-Derived Exosomal MicroRNAs Suppress Myofibroblast Differentiation by Inhibiting the Transforming Growth Factor-beta/SMAD2 Pathway During Wound Healing. Stem Cells Transl. Med..

[23] Liang X., Zhang L., Wang S., Han Q., Zhao R.C. (2016). Exosomes secreted by mesenchymal stem cells promote endothelial cell angiogenesis by transferring miR-125a. J. Cell Sci..

[24] Kurita M., Araoka T., Hishida T., O’Keefe D.D., Takahashi Y., Sakamoto A., Sakurai M., Suzuki K., Wu J., Yamamoto M. (2018). In vivo reprogramming of wound-resident cells generates skin epithelial tissue. Nature.

[25] Sun B.K., Siprashvili Z., Khavari P.A. (2014). Advances in skin grafting and treatment of cutaneous wounds. Science.

[26] Thery C., Witwer K.W., Aikawa E., Alcaraz M.J., Anderson J.D., Andriantsitohaina R., Antoniou A., Arab T., Archer F., Atkin-Smith G.K. (2018). Minimal information for studies of extracellular vesicles 2018 (MISEV2018): A position statement of the International Society for Extracellular Vesicles and update of the MISEV2014 guidelines. J. Extracell Vesicles.

[27] Zhang Y., Hao H., Liu J., Fu X., Han W. (2012). Repair and regeneration of skin injury by transplanting microparticles mixed with Wharton’s jelly and MSCs from the human umbilical cord. Int. J. Low Extrem. Wounds.

