# Supplementary material for: Clinical-Scale Mesenchymal Stem Cell-Derived Extracellular Vesicle Therapy for Wound Healing
Source: Int J Mol Sci. 2023 Feb 21;24(5):4273. doi: 10.3390/ijms24054273 (PMC10001880; doi:10.3390/ijms24054273)
Supplement: Supplementary file 1 [file ijms-24-04273-s001.zip › ijms-2152810-supplementary.pdf]

Clinical scale mesenchymal stem cell-derived extracellular vesicles for wound healing

Jieun Kim, PhD;[1, 2] Eun Hee Kim, PhD;[3] Hanbee Lee, PhD; [1, 2] Ji Hee Sung, MS;[3] Oh Young Bang, MD, PhD.[1, 2, 3, 4]

Supplementary Data

Preparation (3D spheroid culture of WJ-MSC) and isolation of EVs

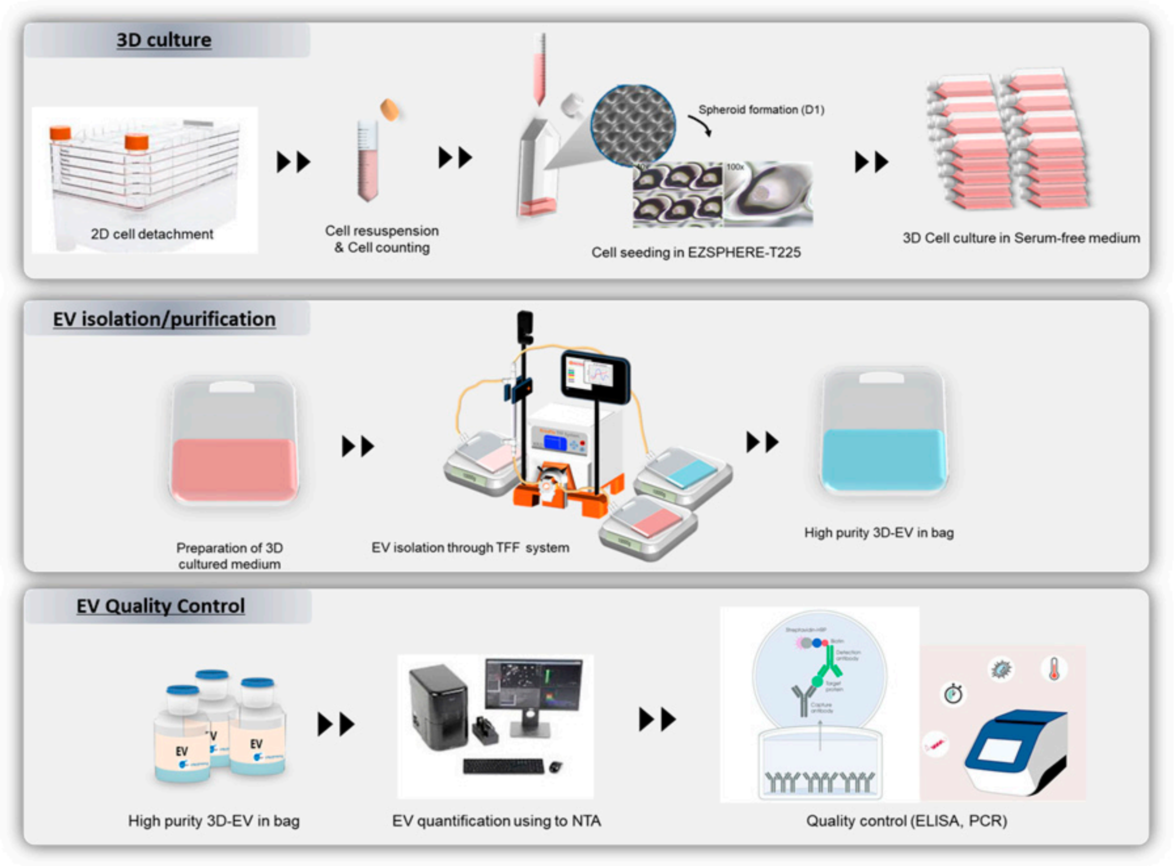

Suppl. Figure S1. Scalable production process extracellular vesicles.

3D-mesenchymal stem cell spheroids were generated in serum-free medium using a micro-patterned well system. EVs were isolated from the culture medium using a combination of filter and tangential flow filtration (TFF) system and measured for concentration and size using nanoparticle tracking (NTA); quality control was done using enzyme-linked immunosorbent assay (ELISA) and quantitative polymerase chain reaction (qPCR).

Characterization of EVs

Nanoparticle tracking analysis (NTA):

The concentrations and size distributions of the isolated EVs were characterised by NTA using a NanoSight NS300 instrument (Malvern, Worcestershire, UK). In the NTA software program, the camera level was set to 14. NTA analysis of EVs after pre-diluted in vesicle-free PBS with EVs showed that PBS did not cause osmotic swelling of the EVs. The sample carrier cells were washed with ethanol at each EV measurement. The mean size and concentration (particles/mL) were calculated by integrating the data from three recordings.

Exoview analysis:

The isolated MSC-EVs were detected for CD81, CD63 and CD9 with ExoView platform (NanoView Biosciences, Boston, MA, USA) according to the manufacture's instruction.

Western blot analysis:

The EV proteins were quantified using a Pierce microBicinchoninic Acid (microBCA) Protein Assay Kit (Thermo Scientific, Rockford, IL, USA) according to the manufacturer's instructions.

Transmission electron microscopy (TEM)

Purified EVs were fixed with 1% OsO_4 dissolved in 0.1 M phosphate buffer (PB) for 2 h. The EM grid was placed Formvar side down on top of the EV drop for approximately 1 min. The grid was removed, blotted with filter paper, and placed onto a drop of 2% uranyl acetate for 15 s. The excess uranyl acetate was removed, and the EM grid was examined and photographed using TEM (JEM-1011, JEOL, Japan).

The direct visualization of EVs was examined by Cryo-transmission electron microscopy (TEM). Carbon grids (Quantifoil, R1.2/1.3, 200mesh, EMS) were made hydrophilic surface with glow-discharged in Pelco EasiGlow system. An aliquot (4 μL) of samples was applied on to the carbon side of EM grid and blotted for 1.5 s with humidity and temperature of 100 % and 4 °C. Then the sample was plunge-frozen into the precooled liquid ethane with Vitrobot Mark IV (FEI, USA). The samples were analyzed by cryo-electron microscope Talos L120C (FEI) at 120 kv.

Exoview analysis

The isolated MSC-EVs were detected for CD81, CD63 and CD9 with ExoView platform (NanoView Biosciences, Boston, MA, USA) according to the manufacture's instruction.

Briefly, EVs diluted with PBS were incubated 24-well plate for overnight with Exoview tetraspanin Chip (EV-TC-TTS-01) arrayed with antibodies against CD81, CD63 and CD9. The antibodies were diluted 1:5000 in PBST with 2% BSA. The chips were incubated with 250 μL of the labeling solution for 2 h. IgG isotype was used as a negative control. After washing, the chips were incubated with Exoview tetraspanin labeling ABs (EV-TC-AB-01) containing the fluorescence-tagged labeling antibodies against CD81, CD63 and CD9. Finally, the chips were imaged with the ExoView R100 reader using ExoViewer 2.5.0 software, and the data were analyzed using ExoViewer 2.5.0 with sizing thresholds set to 50 to 200 nm diameter.

Western blot analysis

Cells and EVs were lysed in radioimmunoprecipitation assay (RIPA) buffer (25 mM Tris-HCl, pH 7.6, 150 mM NaCl, 0.5% Triton X-100, 1% Na-deoxycholate, 0.1% sodium dodecyl sulphate and protease inhibitor cocktail). Twenty micrograms of protein were separated via sodium dodecyl sulphate-polyacrylamide gel electrophoresis and transferred to a nitrocellulose membrane (Bio-Rad, Hercules, CA, USA). The membrane was incubated with primary antibodies against histone H2A.Z, histone H3, lamin A/C, flotillin-1 (1:1000, Cell Signaling Technology, Beverly, MA, USA), or calreticulin (1:1000, ThermoFisher Scientific, Inc., Rockford, IL, USA) overnight at 4°C. After washing with Tris-buffered saline-Tween 20, the membrane was incubated with horseradish peroxidase (HRP)-conjugated secondary antibodies (1:1000, anti-rabbit, Cell Signaling Technology) for 2 h. Proteins were detected using a chemiluminescence substrate from ThermoFisher Scientific, Inc. (Waltham, MA, USA). The labelled proteins were visualised on X-ray film (Agfa, Mortsel, Belgium).

Enzyme-linked immunosorbent assay (ELISA)

ELISAs were performed using commercial kits according to the individual manufacturer's instructions. The following ELISA kits were used: gentamicin (5111GEN, EuroProxima, Arnhem, Nederland), bovine albumin (8100, Alpha Diagnostic, San Antonio, TX, USA), Hsp70 (ab133061, Abcam, Cambridge, UK), CD63, CD9, and CD81 (EXOEL-CD63A-1, EXOEL-CD9A-1, EXOEL-CD81A-1, System Biosciences, Palo Alto, CA, USA), histone H2A.Z. and fibronectin (MBS762894, Mybiosource, San Diego, CA, USA), calreticulin (MBS762894, Mybiosource), synthenin-1 (E4148Hu, BT LAB, Zhejiang, China) and cytochrome C (KHO1051, ThermoFisher Scientific, Inc.). All kits contained standard proteins; thus, the amount of protein and EV counts were determined based on the standard curve from

each kit. A small molecule-containing free protein collected using a TFF system was used as the secretome.

The changes of the characteristics of EVs and EV-miRNAs after repetitive freeze-thawing process.

The number and size of EVs and the level of EV-protein levels were not changed during repetitive freeze-thawing process, up to 3 cycles.

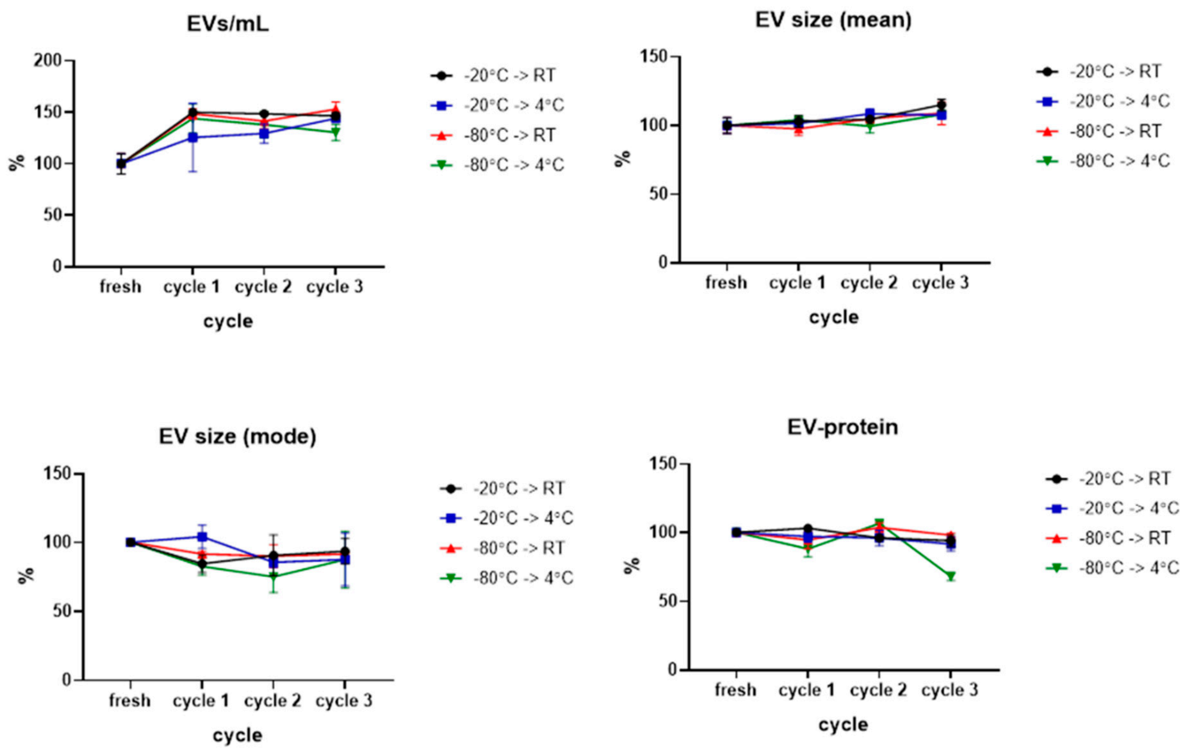

Suppl. Figure S2. Impact of Freeze-Thaw Cycles on EV Characteristics.

MSC-EV microRNA profile

RNA isolation and small RNA sequencing

Total RNA was extracted using the TRIzol^{TM} reagent (Invitrogen, Carlsbad, CA, USA) according to the manufacturer's instructions. RNA quality was assessed on an Agilent 2100 bioanalyzer using the RNA 6000 PicoChip (Agilent Technologies, Amstelveen, The Netherlands), and RNA quantification was performed using a NanoDrop 2000 Spectrophotometer system (ThermoFisher Scientific, Waltham, MA, USA).

To evaluate the expression profiles of the miRNAs in the EVs, small RNA sequencing was performed. For the control and test RNAs, library construction was performed using the NEB Next Multiplex Small RNA Library Prep kit (New England BioLabs, Inc., Ipswich, MA, USA) according to the manufacturer's instructions. Briefly, for library construction, 1 μg total RNA from each sample was used to ligate the adaptors and then cDNA was synthesised using reverse-transcriptase with adaptor-specific primers. Polymerase chain reaction (PCR) was performed for library amplification, and libraries were purified using the QIA quick PCR Purification Kit (Qiagen, Inc., Hilden, Germany) and AMPure XP beads (Beckman Coulter, Inc., Brea, CA, USA). The yield and size distribution of the small RNA libraries were assessed using the Agilent 2100 Bioanalyzer instrument for the High-sensitivity DNA Assay (Agilent Technologies, Inc., USA). High-throughput sequences were produced using the NextSeq500 system as single-end 75 bp sequences (Illumina, San Diego, CA, USA).

miRNA highly expressed in our MSC-EVs

Top 150 known miRNAs detected in the EVs isolated from the 3D-cultured MSCs by small RNA sequencing. Normalised read counts are shown (n=2) (Suppl. Figure S3A). Comparing the exosomal RNA-Seq results between WJ-3D-EVs and WJ-2D-EVs can provide important information about the wound-healing process (Suppl. Figure S3C). The miRNA and cargo were isolated from EV and then analyzed to identify their role in wound healing. The results showed that the miRNA containing EV and its cargo regulate various signaling pathways associated with wound healing and tissue regeneration, including cell proliferation, migration, and angiogenesis (Suppl. Figure S3B).

A

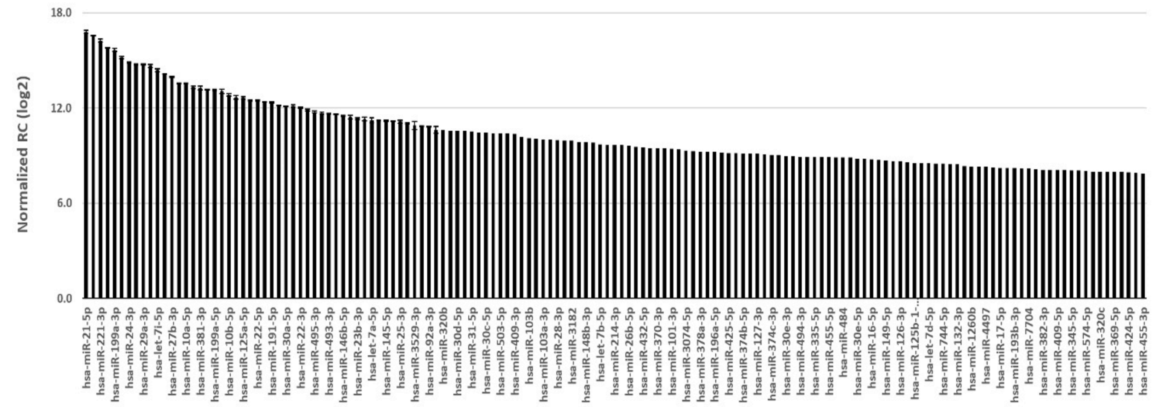

B

MicroRNA	Target	Effects in wound healing	Ref.
miR-21-3p	PI3K/Akt, ERK1/2	PTEN and SPRY1↓	[1]
miR-21-5p	Wnt/β-catenin signaling	miR-21-5p loaded MSC-EVs accelerated diabetic wound healing by increasing re-epithelization, collagen remodeling, angiogenesis and vessel maturation *in vivo*.	[2]
miR-21, 23a, 125b, 145	Inhibit TGF-β2/SMAD2 pathway	MSC-EV microRNAs suppressed myofibroblast formation by inhibiting excess alpha-smooth muscle actin and collagen deposition and inhibited excessive scar formation caused by myofibroblast aggregations.	[3]
miR-125a	Notch signaling pathway (suppress DLL-4, IL-6)	MSC-EVs transfer miR-125a to endothelial cells and promote angiogenesis and tissue repair	[4]
miR-126-3p		miR-126-overexpressing MSC-EVs in heal full-thickness skin defects in a diabetic rat model	[5]
557 miRNAs, including miR-555, 1972, 3907, etc.	TIMP-1 and TGF-β1	miR functions for fibroblast proliferation, ultraviolet protection, collagen biosynthesis, DNA repair, cell aging.	[6]

C

Reported effects	miRNA symbol	Fold change
TNF-α inhibition	hsa-miR-181a-5p	1.330
IL-1β inhibition	hsa-miR-30a-3p	1.321
	hsa-miR-328-3p	1.414
	hsa-miR-149-5p	1.261
COX-2 inhibition	hsa-miR-101-3p	2.246
	hsa-miR-146a-5p	2.653
	hsa-miR-181c-3p	1.403
Migration and proliferation of keratinocytes	hsa-miR-31-5p	1.340
	hsa-miR-205-5p	1.461
Migration and proliferation of fibroblasts	hsa-miR-132-3p	3.063
Angiogenesis	hsa-miR-146a-3p	3.373
	hsa-miR-27a-3p	3.191
	hsa-miR-132-3p	3.063
	hsa-miR-146b-5p	2.901
	hsa-miR-146a-5p	2.653
	hsa-miR-98-3p	2.488
	hsa-miR-22-5p	2.471
	hsa-miR-301b-3p	2.369
	hsa-miR-194-5p	2.309
	hsa-miR-101-3p	2.246
	hsa-miR-24-2-5p	2.200
	hsa-miR-495-3p	2.145

Suppl. Figure S3. *miRNA highly expressed in our MSC-EVs have therapeutic effects in wound healing* (A) The top 150 known miRNAs detected in exosomes isolated from 3D-cultured mesenchymal stem cells by small RNA sequencing. (B) The target of EV-containing miRNA and cargo related to wound healing mechanisms. (*C) Exosomal RNA-Seq results between WJ-3D-EV vs WJ-2D-EV*

MSC-EV and Wound Healing Biomarker Gene Expression Analysis

Compared with MSC-EV treated fibroblast cells and the control groups, results indicated that during the wound healing process in fibroblast and keratinocyte cells, the expression of VEGF-A, Wnt, and *Adenosine related pathway activation (PI3K/AKT)* in the MSC-EV treated group was significantly higher than in the control group. These findings suggest that MSC-EV treatment may promote wound healing by regulating these specific signaling pathways. These

findings suggest that EV-contained miRNA and cargo play a key role in regulating the wound-healing process through the modulation of these signaling pathways. Further studies are needed to fully understand the mechanism of action and the potential therapeutic applications of EV-contained miRNA and cargo in wound healing.

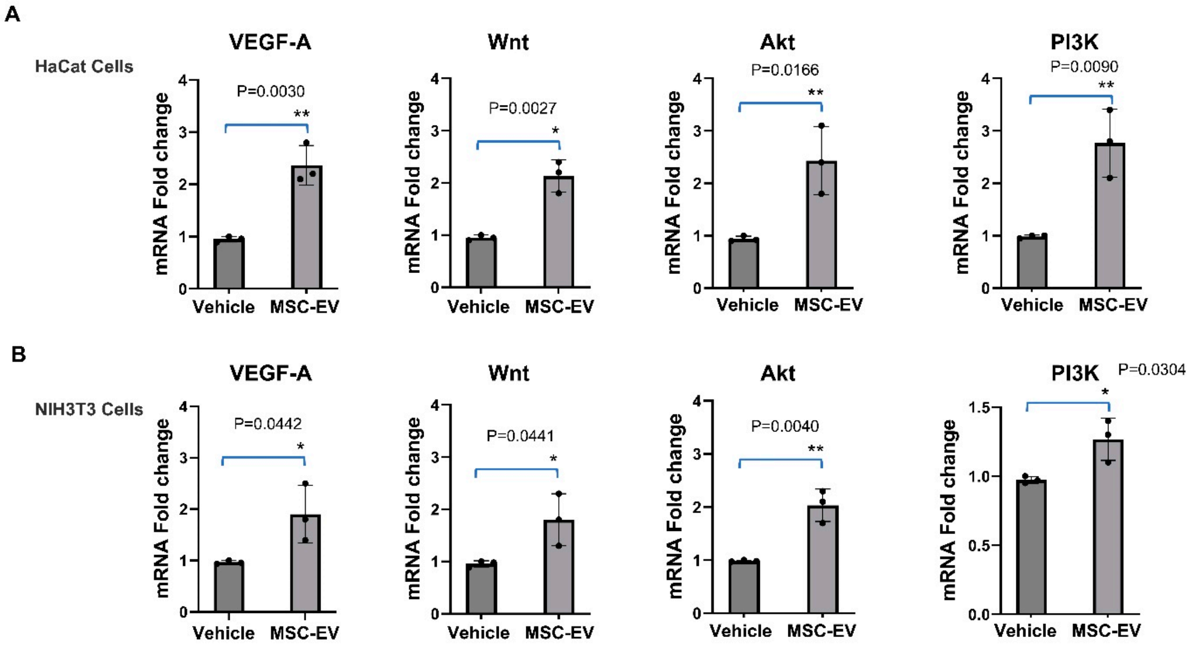

Suppl. Figure S4. The mRNA expression of biomarkers related to the wound healing mechanism by MSC-EV was analyzed using real-time polymerase chain reaction analysis of MSC-EV treated cells and control. (A - B) The asterisk represents a significant difference in expression (**$p < 0.01$, and *$p < 0.05$). Error bars indicate standard deviations.

References

1. Hu, Y.; Rao, S. S.; Wang, Z. X.; Cao, J.; Tan, Y. J.; Luo, J.; Li, H. M.; Zhang, W. S.; Chen, C. Y.; Xie, H., Exosomes from human umbilical cord blood accelerate cutaneous wound healing through miR-21-3p-mediated promotion of angiogenesis and fibroblast function. *Theranostics* **2018,** 8, (1), 169-184.
2. Lv, Q.; Deng, J.; Chen, Y.; Wang, Y.; Liu, B.; Liu, J., Engineered Human Adipose Stem-Cell-Derived Exosomes Loaded with miR-21-5p to Promote Diabetic Cutaneous Wound Healing. *Mol Pharm* **2020,** 17, (5), 1723-1733.
3. Fang, S.; Xu, C.; Zhang, Y.; Xue, C.; Yang, C.; Bi, H.; Qian, X.; Wu, M.; Ji, K.; Zhao, Y.; Wang, Y.; Liu, H.; Xing, X., Umbilical Cord-Derived Mesenchymal Stem Cell-Derived Exosomal MicroRNAs Suppress Myofibroblast Differentiation by Inhibiting the Transforming Growth Factor-beta/SMAD2 Pathway During Wound Healing. *Stem cells translational medicine* **2016,** 5, (10), 1425-1439.
4. Liang, X.; Zhang, L.; Wang, S.; Han, Q.; Zhao, R. C., Exosomes secreted by mesenchymal stem cells promote endothelial cell angiogenesis by transferring miR-125a. *Journal of cell science* **2016,** 129, (11), 2182-9.
5. Tao, S. C.; Guo, S. C.; Li, M.; Ke, Q. F.; Guo, Y. P.; Zhang, C. Q., Chitosan Wound Dressings Incorporating Exosomes Derived from MicroRNA-126-Overexpressing Synovium Mesenchymal Stem Cells Provide Sustained Release of Exosomes and Heal Full-Thickness Skin Defects in a Diabetic Rat Model. *Stem cells translational medicine* **2017,** 6, (3), 736-747.
6. Choi, J. S.; Cho, W. L.; Choi, Y. J.; Kim, J. D.; Park, H. A.; Kim, S. Y.; Park, J. H.; Jo, D. G.; Cho, Y. W., Functional recovery in photo-damaged human dermal fibroblasts by human adipose-derived stem cell extracellular vesicles. *J Extracell Vesicles* **2019,** 8, (1), 1565885.
